# Society Meetings

**Published:** 1914-09

**Authors:** 


### SOCIETY MEETINGS

Brief reports and announcements of meetings of societies of Western New York, and those of general scope, are requested from Secretaries. Copy should be on hand the fifteenth of the month. Full transactions will be published at cost of composition.

### American Proctologic Society.

Sixteenth Annual Meeting, held at Atlantic City, June 22 and 23, 1914. President, Jos. M. Mathews, Louisville; Vice-president, Jas. A. MacMillan, Detroit, in the chair. Officers elected for the ensuing year: President Louis J. Krouse, Cincinnati; Vice-president, Collier F. Martin, Philadelphia; Secretary-Treasurer, Alfred J. Zobel, San Francisco. The place of meeting for 1915 will be San Francisco, Cal. Exact date and headquarters will be announced later. The following were elected Associate Fellows of the Society: Dr. Wm. H. Axtell, Exchange Block, Bellingham, Wash.; Dr. Rolla Camden, 915 Avenue of the Presidents, Washington, D. C.; Dr. Descum C. McKenney, 1250 Main street, Buffalo N. Y.

Extracts From the Report on Proctologic Literature From March, 1913, to March, 1914. Samuel T. Earle, Baltimore.

In his review of Proctologic Literature from March, 1913 to March, 1914, he quotes from many authors giving the salient points from each of their papers.

Coccygodynia: A New Method of Treatment by Injections of Alcohol. Frank C. Yeomans, New York City.

The diagnosis is established by a thorough examination, both general and local. Local examination is made by inserting the index finger into the rectum and palpating the coccyx between it and the thumb outside. The soft parts intervening between the coccyx and anus are now compressed and the point of maximum tenderness is thus located, usually just beyond the tip of the coccyx. Proctoscopy rules out rectitis.

The prognosis hitherto has been better in the traumatic cases than in those of frank neuralgia or neuritis. The writer confidently predicts that the treatment proposed will render the latter equally amenable to treatment.

The writer proposes a treatment based on the suggestion of Schlosser in 1907, of injecting 70 to 80 per cent. alcohol in sensory nerves, thereby causing their degeneration as practised with marked success in trifacial neuralgia.

The technique is simple and can be carried out in the office under strict aseptic precautions. The patient with empty bowel is placed on a table in the Sims' position and the skin about the coccyx painted with tincture of iodine. A 2 c. c., Luer or similar syringe is filled with 80 per cent. alcohol and armed with a two inch needle. The right index finger is now inserted into the rectum and the point of maximum tenderness is determined by counter pressure with the thumb outside. Maintaining the finger in the rectum to guard against puncture and as a guide, the needle is introduced through the midline directly to the painful spot, and 10 to 20 minims of solution are injected slowly.

The needle is withdrawn and its puncture sealed with collodien. The pain from the injection lasts a few minutes and is followed by a dull ache which may last a day or two. From three to five injections are usually required at intervals of about one week.

The writer reports seven cases, all women, treated from two months to four years ago. They required three, four or five injections each at intervals of about one week. Relief was prompt and complete and all the patients have remained well.

The Technique of the Perineal Operation for Cancer of the Rectum. J. A. McMillan, Detroit.

In every case a preliminary colostomy must be considered imperative. The colostomy provides the only means of discovering whether a radical operation is justifiable or not, supplies physiologic rest for the affected part, and later provides for aseptic conditions in the surgical field.

After thorough divulsion a circular incision is made at the muco-cutaneous line and carried up to the lower surface of the Levator Ani. Most of the dissection can be done by the fingers. It is not necessary to destroy the external sphincter. This step of the operation exposes a circular area of the Levator Ani about an inch and one half wide. Before proceeding further the hemorrhage should be controlled and the location of affected glands determined.

The next step of the operation includes the division of the Levator Ani and the removal of lymphatic glands.

The peritoneum may be entered anteriorly and separated laterally which will leave the mesosigmoid as the only attachment of the bowel. This should be divided as far from its colonic attachment as possible in order to secure the retention of a good vascular supply for the proximal end of the bowel after the excision.

When the gut can be drawn down sufficiently to permit the excision of the affected portion and the attachment of the lower edge of the mucous membrane to the skin, excision is done and the sutures placed. Free drainage is necessary. The colostomy is not closed until the patient has been up and about for several weeks.

Myasthenia Gastro-Intestinalis. V. Lee Fitzgerald, Providence, R. I.

By the term "myasthenia gastro-intestinalis" is understood a weakness of the muscles of the abdomen, stomach, intestines, and their supporting ligaments, with a consequent downward displacement of any or all of the viscera.

Many patients suffering from myasthenia in its different forms are in danger of having suspensory or other operations performed upon them, whereeas the intestinal stasis can be entirely removed by medical measures and the baneful effects of the underlying ptosis entirely removed.

The general aim in the treatment is the relief of the stasis, and the restoration of the prolapsed viscera to as near their normal position as possible.

The success in the treatment of these patients depends not only upon the relief of stasis, but also upon the patient's active and persistent co-operation.

For the past two years the writer has been treating cases of myasthenia as follows: The patient is given a thorough examination, including that of the gastric contents, urine and faeces. In case of myasthenia of the stomach with dilatation and prolapse the patient is put to bed and fed through a duodenal tube six or seven times a day, depending upon the amount of food needed to nourish the patient. This gives the stomach a complete rest, and it comes up into normal, or nearly normal, position in from ten days to two weeks.

Further Observations on Pruritus Oni: Its Probable Etiological Factor; Results of Treatment. (A fourth report, based on results of original research.) Dwight H. Murray, Syracuse, N. Y.

In this report on the fourth year's work of original research on pruritus ani, the author finds there is not much more to give to the profession beyond the confirmation of the work of previous years. He has yet no reason to doubt his claims for the infection theory of pruritus ani.

Twenty new cases have been examined during the past year. In all but two of these streptococcus fecalis has been demonstrated.

It has been found that occasionally the bacterial growth seems to be so lacking in strength that it is difficult to obtain an autogenous vaccine. It is not known why this is so unless it is owing to the very low grade inflammation produced by germs not so active as those found in many other infections.

During this year two cases were treated by other physicians who tried to follow his technique, but in neither case was improvement manifest notwithstanding that streptococci were found present by the author's bacteriologist and although the same quality of vaccines were used. With the consent of their physician the author took up the treatment. Improvement was marked. The only point of difference in the technique that he could discover was that the others injected the vaccine deep into the muscle instead of directly into the skin or immediately beneath it.

During the past year the author has had additional proof that the itching does not extend appreciably above the white line of Hilton. He has also had continued confirmation of his previous statement that the moisture found upon the parts is not a discharge from the rectum.

This past year's work again shows that other rectal diseases are not present regularly with pruritus ani, and the belief is confirmed that they are coincidental instead of etiological.

No unfavorable sequelae arose from the vaccine injections. there is now no hesitation in running the dose up to two billion or more dead bacteria. One injection resulted in formation of a jelly-like material in the tissue but this was absorbed. Some time ago a similar swelling was opened and found to be sterile, and no trouble has resulted.

A Report of Cases of Pruritus Ani Treated With Carnotite.
Samuel T. Earle, Baltimore, Md.

Carnotite, a radio-active mineral, was used in the treatment of eight cases of pruritus ani and was found to be a very satisfactory palliative remedy.

Treatment of Amebic Dysentery by Emetine Hydrochloride.
Alfred J. Zobel, San Francisco, Cal.

He reports two interesting cases in which the disease was present in one individual for ten, and in the other for fourteen years. Under the influence of emetine, within two or three days amebae, blood, mucous, froth, and foul odor disappeared from the dejections and their number greatly decreased; the racking tenesums, bearing down feeling in the rectum, the colic, and the abdominal tension, discomfort, and gurgling absolutely ceased. Proctoscopic examinations revealed the favorable influence of the drug upon the amebic ulcerations. No amebecidal irrigations were employed. He advises that emetine should be given for at least three or four months at intervals before the patient should be considered free from the possibility of a recurrence, even though he is clinically cured and the amebae cannot be longer found in the stools.

Amebic Dysentery and Its Treatment. Dr. Wm. M. Beach, Pittsburgh, Pa.

(1) Amebic dysentery in the early stages may be cured with emetine. (2) In cases somewhat advanced emetine is efficacious and at least clinically curative. (3) The use of the duodenal tube, through which to introduce solutions of emetine to any portion of the intestinal tract, should receive trial and consideration. (4) For rapid cure, and control, cecostomy or appendicostomy is the best measure in advanced and chronic cases. (5) Direct irrigation from above is superior to rectal injections, in that it is less painful and more thorough. (6) The appendix should be removed in most cases of amebic dysentery. (7) The so-called specific emetine can be easily applied in weak solutions.

The Pathologic Sigmoid Colon and Its Surgery. L. J. Hirschman, Detroit, Mich.

Studies with the fluoroscope and the sigmoidoscope have shown that true prolapse and invagination of the sigmoid colon into the rectum is not an uncommon condition. The author advocates shortening the mesentery of the sigmoid by attaching the mesentery of the invaginated or prolapsed portion to the root of the mesentery of the descending colon.

In a number of cases of obstruction to normal defecation, this obstruction will be found in women who give a history of a disturbed puerperium. Radiographic studies of these patients who give a history of chronic obstipation accompanied by pain and marked tenderness in the left lower abdominal quadrant and the region of the womb and broad ligaments, more often the left, show the presence of adhesions which angulate, displace or bind down the sigmoid. The cure of this condition involves the relieving of the adhesions and the grafts, or the excision or short circuiting of the sigmoid. Another class of adhesions of the sigmoid seriously obstructing covering of raw areas with omental, epiploic or mesenteric defecation is caused by adhesions to the abdominal wound following laparotomy.

Hypertrophy or redundancy of the sigmoid colon is another pathological condition which has not infrequently been met with. When the walls of the bowel contain a large proportion of unyielding fibrous tissue, short circuiting is insufficient and excision is indicated.

In malignant growths of the sigmoid colon, excision with immediate anastomosis is the ideal indication.

When inoperable it is the author's practice to always make the colostomy in the median line. This in done for the following reasons: First, the median incision is the best for exploratory purposes. Second, one has the choice of any part of the colon in the making of the colostomy. Third, one gets just as good adhesion and union, with no more liability to hernia, as in the side. Fourth, the patient is better able to cleanse and dress the colostomy in the median line. Fifth, it takes the colostomy opening away from the neighborhood of the iliac crests, and allows of the better fitting of retention apparatus and colostomy shields. Sixth, control of a medial colostomy is just as satisfactory as the lateral.

The author has found no difficulty in securing colostomy control by using a small rubber catheter in the mesenteric opening beneath the spur and encircling the upper limb of the colostomy with this catheter, drawing it just snug enough that the mucous surfaces oppose. The catheter is held in this position by a seraphine snap and is released by the patient when he wishes to defaecate or expel flatus.

Membranous Enteritis—Mucous Colic. Dr. S. G. Grant, New York.

The assayist explained that myxorrhea coli was a symptom complex characterized by constipation, abdominal pain, uneasiness or soreness and the periodic evacuation of jelly-like strips or casts of tenacious mucus on the one hand or colic on the other hand suggested that all mucous discharges as Myxorrhea Colic with which understanding the former is called Myxorrhea Membrancea and the latter M. Colica. The writer conceded that either type of myxorrhea coli may be secondary to neurogenic disturbances but strongly maintained that M. Membranacea and M. Colica are frequently produced by many other conditions and diseases, medical and surgical, several of which may be factors in the same case. He had often known these conditions to be caused by psychic, neurogenic, gastrogenic and enterogenic disturbances, abenoidism, thyroid disease, impaired metabolism, abnormal menstruation, affections of the heart, liver and pancreas, inflammatory and ulcerative lesions (colitis), helminths, foreign bodies, prolonged or irritating colonoclysis, various lesions which induce chronic intestinal obstruction and led to coprostasis and autointoxication and other ailments which cause the hypersecretion or retention of mucus. The writer had observed patients who suffered at first from myxorrhea membranacea and later M. colica where the mucus became inspisated irritating and excited enterospasm.

The writer maintained that the diagnosis was easy in uncomplicated cases and that Myxorrhea Membrancea could be recognized by its Symptom Complex, obstinate constipation, uneasiness and soreness or pain in the lower left abdominal quadrant and the periodic discharge of strips, casts, or jelly-like masses of mucus, and that where subsequent of these manifestations and in the absence of signs pointing to intestinal obstruction from other causes colic suddenly supervenes, one is justified in making a diagnosis of myxorrhea colica.

The essayist discountenanced a routine treatment in these cases and advised holding curative measures in the abeyance until the acute symptoms subsided.

The removal or correction of kinks, twists, strictures, invaginations, adhesions, pericolic membranes and other lesions obstructing the bowel or causing stasis, effected a cure in many of the writer's cases and he rarely found the bowel sufficiently incapacitated to require resection, exclusion, or the establishment of an artificial anus.

In conclusion the writer stated that myxorrhea membranacea and M. Colica were common affections and more frequently responded to surgical treatment than the literature of the subject would indicate.

Peri-Rectal Gumma: Report of Two Cases. Alois B. Graham, Indianapolis.

The subject owes a great deal of its interest to its rarity. The author reports two cases which are rather unique. They were seen within twenty- four hours of each other, and both presented a typical peri rectal gumma, in that no lesion of any kind could be detected in the rectum of either patient.

The author's conclusions are that Peri rectal gummata are rare. The two cases reported are unique and of interest in that both were typical examples of peri rectal gummaeta. In both cases the gumma was seen in its early or vascular phase. In one case it appeared 23 years after the initial lesion; in the other case it appeared three years following the syphilitic infection. 'Both gummata were painless to palpation and fluctuation was detected in both. An error of diagnosis in one case was responsible for the incision and subsequent suppuration which followed. In the other case no incision was made and suppuration did not occur. No demonstrable rectal lesion could be discovered in either case. The induration in both cases disappeared rapidly under antisyphilitic medication. No fistula resulted in either case.

Anal and Rectal Growths of Benign or Doubtful Character. Dr. T. Chittenden Hill, Boston.

Hill states that in a series of 3000 rectal cases previously reported there were 49 benign and 76 malignant growths of the rectum. The large majority of these tumors were characteristic and the differential diagnosis was easily made. A few malignant growths seen in an early stage, and some unusual benign types associated with ulceration, were of such a nature that the exact diagnosis was not easily determined.

The writer emphasized the fact that the operative measures to be employed differ radically in each of these conditions. An excision of the rectum is necessary for the malignant cases, a simple local excision is all that is required for the benign growths, where an incision and drainage will suffice for the abscesses and fistulae. Therefore, a doubtful case cannot be treated as a breast case in which a complete amputation for a benign growth may be justified. In the case of the rectum there is not alone mutilation but a high mortality and a serious impairment of function as well to be considered. Furthermore, the removal of a specimen of a suspected tumor is not now approved and this complicates the problem still more.

The histories of several cases which illustrate the doubtful nature of some border line conditions occasionally found in the rectum are cited. They tend to show that aside from benign growths some of which have many of the characteristics of malignancy, there are certain abscesses which develop in the loose cellular tissue of the rectro-rectal and pelvi-rectal spaces which are even more suspicious. These indurated, irregular swellings bulging into the rectal ampullae at first resemble very closely the sensation imparted to the finger in malignancy. A little later they become soft and fluctuation is perceptible when all doubt as to their nature is removed. The sinus from an old fistulae occupying these same spaces is apt to be much more perplexing than an abscess. As the slow process goes on the rectal wall is crowded into the lumen of the bowel and assumes an irregular, indurated outline which is very suggestive of cancer. Other conditions of similar doubtful character such as gummatous growths and tubercular ulceration are also discussed.

Retrorectal Infections. Collier F. Martin, Philadelphia.

Martin reviews the histories of sixty-seven cases. In addition to the infection of the retrorectal space many of the cases also had involved the pelvirectal and ischiorectal spaces. Some of the more chronic cases were complicated with stricture of the rectum and multiple fistulae.

Eighty-five per cent. of the infections occurred in males. External traumatism was not a factor in this series of cases. The author holds that most of these infections originate from internal traumatism, associated with some condition which lowers the resistance of the individual to pyogenic infection.

Pulmonary tuberclosis appears to be the most constant factor in thus lowering the resistance. Twenty-one per cent. died from tuberclosis at varying periods, either after examination or operation.

Forty-three per cent. of the cases are noted as having pulmonary tuberclosis more or less advanced.

Of the fifty-five cases operated upon, thirty-three were cured. These present sixty per cent. of the operative cases, or nearly fifty per cent. of the total number examined.

In nearly half of the cases the original abscesses had opened posteriorly, either between the sphincters or at the anorectal line. Pain was not a prominent symptom.

The methods of incision applicable to the various complicating conditions are briefly outlined.

The author lays great stress upon the seriousness of infections, and upon the necessity of the prolonged watchful after-treatment.

While the prognosis as to both complete recovery of the local condition and the general health, as well as to the preservation of the sphincter control, should be guarded, careful after-treatment and prolonged observation will result in saving a large proportion of these really serious cases.

An abbreviated history of the findings in the entire sixty-seven cases is given.

Hemorrhoids; Their Treatment. Dr. J. Rawson Pennington, Chicago.

Dr. Pennington states that clinically hemorrhoids should be classified:

(1) According to their location.

(2) According to their structure.

According to their structure they are divided into, (a) those containing fluid blood, (b) those containing clotted blood, (c) those containing both fluid and clotted blood, and (b) those consisting of "skin tabs" or folds of skin.

Most hemorrhoidal cases can, be operated on under some form of local anesthesia. He operates on 90% of his cases by blocking the field of operation. The cocaine is usually employed in the strength of from ¼ to ½ of 1%. The quinine and urea in from ¼ of 1% to 1% solution. Sometimes he combines the solutions, the cocaine being used for its immediate effect and the quinine and urea for prolonging the anesthesia.

During the last 20 years he has given a fair trial to a number of methods advocated which promised a reasonably good result, including the ligature, the clamp and cautery, Whitehead, injection, suturing and other methods which unite tissue in mass, and has come very definitely to the conclusion that by far the best way of treating this condition is by the excision or enucleation method.

The operative procedure should have for its object the removal of the cause of the tumefaction. The treatment for each type of hemorrhoid should be practically the same. This should consist in removing an ellipse from the tumor-like formation and in the case of the thrombotic pile turning out the clot, and in that of the internal variety the varicosity and allowing the blood to escape, and in the fleshy pile of dissecting out the excess of tissue.

Hyperplastic Tuberculosis of the Colon. J. M. Frankenburger, Kansas City.

The writer declared that this form of tuberculosis of the intestine differs from other forms of intestinal tuberculosis, inasmuch as it is amenable to operative interference. It is generally a local and primary lesion and is characterized by the formation of tumor masses composed of fibrous and tuberculous granulation tissue in the walls of the bowel. Primarily there is no involvement of the mucous membrane, but on account of the narrowing of the gut the irritation caused by the passage of feces may produce ulceration.

Symptoms are slight, constipation and diarrhoea sometimes alternating. Later the symptoms are those of gradually increasing intestinal obstruction. Differential diagnosis is between sarcoma, carcinoma, syphilis, and chronic appendicitis with adhesions.

Treatment is purely surgical. If possible the entire growth should be removed, but failing in this a short circuiting operation should be performed to relieve the obstruction.

Two cases are reported with successful operations.

Pseudo-Intestinal Stasis and Real Intestinal Stasis, Demonstrated Roentgenologically. Arthur F. Holding, New York.

Attention is called to many anomalies of visceral position and progress of the bismuth meal that have been interpreted as pathologic, and which are really physiologic or anatomic anomalies and completely compatible with health, laying especially stress upon the fact that the ileum enters the caecum normally at an angle, and unless associated with proximal distension, a diagnosis of Lane's kink is not justified.

He emphasized the point that delayed progress of the bismuth meal is not significant of obstruction unless it is more than 6 hours behind the normal schedule and associated with marked distension of the viscus proximal to the locus of obstruction. Proximal distension with obstruction to the bismuth column are the two cardinal diagnostic points of real intestinal stasis. Intestinal obstruction, due to tumors, is much easier to diagnose than intestinal stasis, because the defect in the bismuth shadow made by the tumor is more definite than that made by adhesions, veils, or membranes.

Local Treatment of Anal Fissure. Jas. A. Duncan, Toledo.

The writer describes a treatment for anal fissure which he has employed successfully for the past thirteen years. The fissure is brought into view by separating the folds, and the surface is lightly curetted, then thoroughly dried, and a drop of collodion applied. This takes only a moment or so. A recent ulceration requires but a single application. A sharp stinging pain lasting for only a few minutes is caused, and then the patient is left perfectly comfortable.

Some Unusual Phases of Sigmoidoscopy. Ralph W. Jackson, Fall River.

The diagnostic value of the sigmoidoscope has been the topic of much writing, and is increasingly appreciated by hospitals, but much less so by the profession and insufficiently in medical teaching. Explicit statements of its considerable therapeutic uses are not found in German, American or English literature. The instrument enhances the extent and accuracy of recto-sigmoidal therapeutics, and specifically it facilitates the use of certain other instruments, topical applications, the relief of high impaction, and the treatment of stricture and many other lesions. Serious trauma from the sigmoidoscope is more liable to happen than some authorities admit, as illustrated by three cases of intestinal perforation cited from the German. Two personal cases are detailed, where the patients were in serious condition from occlusion of the bowel, but were relieved and saved by sigmoidoscopy done with diagnostic intent only. Pelvic visceroptosis, hypermobility of the sigmoid, and the fixed and open rectal ampulla beneath predispose to invaginations and angulations which are fairly frequent in mild and chronic form, and are potentially dangerous as a source of acute obstruction. Sigmoidoscopy, properly conducted, empties the pelvis by gravity (due to the position assumed) by intelligent introduction of the instrument and by the air pressure admitted through it, and therefore tends to undo such intestinal malpositions. The occlusion in the two cases related was unexpectedly relieved, and doubtless in this way. Greater prevalence in the use of the sigmoidoscope would bring to light a field for deliberate therapeutic use of the instrument along these lines.

Crude and Careless Diagnostic Methods, and Results of Same, in Some Recto-Colonic Condition. Jno. L. Jelks, Memphis.

Reference is made to cases operated on for appendicitis, which disease may be an extension of an infection and inflammation originating in the rectum or colon.

Cases are cited to show the frequency and at all times the liability of mistaking a condition for an infection or ulceration of the colon, specific in character, when a coloptosis or pericolic membranes, or both, were the true etiologic factors. Stress is laid on the importance of urinalysis, microscopic examinations and X-ray in rectocolonic cases.

A harder nodular calcareous degeneration of the outer one of the mamma has been observed as a sequence of coloptosis and defective drainage. In another case, in which was found a cecum cradled in pericolic membranes, and a coloptosis, a duodenal ulcer was diagnosticated. In this case the urinalysis, the history, and general toxic appearance of the patient, pointed to true etiology.

Case reports are given in which diarrhea was the dominant symptom, though impactions, pericolic membranes, and ptosis were the true etiology.

The author calls attention to his prior reference to, and work of establishing the importance of conserving the ilio-cecal valve; also to the syphonage of a ptosed colon after short circuiting operations, which he accomplished by a second anastomosis between the blind colon and the sigmoid or rectum below the first anastomosis.

Importance is claimed for a microscopic examination of the intestinal contents of patients who suffer from attacks of appendicitis, and of the contents of the removed appendix; and the author insists that in the event that pathogenic amebae are found appendico-cecostomy should be performed instead of appendectomy.

The author refers to his observation of quite marked congestion of blood in the visceral vessels themselves in these cases of ptosis and defective intestinal drainage.

The author refers to the frequency with which he encounters cases of inoperable cancers of the rectum and intestines, the neglect of which is most often due to the fear of examination of those suffering with symptoms in the regions referred to.

Reference is made to the operation of appendico-cecostomy as being practically free of danger to life. In his opinion this operation would save almost every life that is today caused by the ravages of amebic colitis.

Abscess Originated in a Pilo-Nidal Sinus. Louis J. Krouse, Cincinnati.

The writer states that a pilo-nidal sinus is a congenital defect due to a faulty development of the foetus. It is usually located in thé median line over the coccyx or the sacrum. Inflammation developing in the sinus is followed by burrowing of pus into the neighboring tissue. Inflammation of this sinus must be differentiated from necrosis affecting the sacral or coccygeal bone; from abscess originating in the sebaceous gland of this region; and from true fistula-in-ano. The treatment consists in the complete obliteration of the walls of the sinus.

Abnormalities of the Colon, as Seen With the Roentgen Ray: Lantern Slide Demonstration. W. I. LeFevre, Cleveland.

The entire alimentary tract can now be successfully examined with the X-ray, some parts more readily and successfully than others, according to the degree of satisfaction arranging themselves in the following order: Colon, Stomach, Oesophagus, Small Intestine. Two methods of examination are used. First, Roentgenoscopy, which is the examination with the fluoroscope. Second, Roentgenography, the making of X-ray plates. The Colon is also accessible from either end, that is it can be examined by following the bismuth meal through from the stomach, or by giving an opaque enema of barium sulphate. In the former method the motor phenomena of the colon can be observed, in the latter the size, position and contour can be seen.

The action of atropin, adrenalin, pilocarpin and physostigmine as affecting the action of the bowel is briefly discussed.

The normal colon is described in detail, with radiographs showing different types. Many vary from the "ideal" type and still are normal for that individual.

Abnormalities of the colon may be produced by congenital defects, disease or injury to the bowel proper, from pressure, constriction or relaxation of other organs in close proximity. Coloptosis, owing to its frequency and importance is first discussed with radiographs showing these conditions. Other abnormalities consist of stenosis, malignant growths, tuberculosis, kinks, twisting, hernias, diverticulae, and megacolon or Hirschprung's disease. All these conditions can be recognized by aid of the X-Ray.

Some Problems Before the American Proctologic Society. J. A. MacMillan, Detroit, Mich.

The writer recommends that a cancer committee be appointed to take charge of this work.

---

**The Gross Medical Club** held its August meeting at the residence of Dr. A. L. Phelps, East Aurora, on Friday, the 7th. The paper, Pabulum, was read by Dr. W. W. Britt of Tonawanda. This paper, although to a great extent historical in nature was a masterly consideration of the subject and was freely discussed by Drs. King, Tyler, Baker, Phelps and others. (Will be published in the near future.)

Cases were reported as follows:

Dr. Geo. F. Cott. The case of a lady who two years ago he operated on for mastoiditis, and although she has not been exactly well for the past six months, complained of practically no symptoms that pointed to the condition revealed at the post mortem. She died suddenly and careful examination showed five distinct abscesses of the brain, a condition rarely found: brain abscess and no symptoms.

Dr. J. Henry Dowd. A man 37 in April, 1913, suddenly seized with giddiness, muscular twitchings, body contortions, yelling, etc., to be followed by unconsciousness. Diagnosis hysteroepilepsy and suggestion made that there was some local condition acting as a cause. Except for the use of valerian; nothing was done further, the patient neglecting to call. In June, 1913, another attack occurred which I did not attend him for, but he presented himself for examination later. Further than a history of recurring herpes preputialis, and a slight tenesmus at times, no further information was in evidence. Examination revealed a stricture, quite tight, in the deep urethra, an operation was advised and performed inside of 24 hours. No hystero-epileptic seizures has occurred in 16 months.

Following the regular business, dinner was served at the Roycroft Inn, after which, "the Sage of East Aurora," Elbert Hubbard, gave the members a 15 minutes talk.

---

**The American Roentgen Ray Society** will meet in Cleveland at the Hotel Hollenden on September 9th to 12th inclusive, 1914. The program promises to be of unusual interest and value, and includes a paper by Dessauer of Frankfort, on the subject of artificial production of gamma rays; Coolidge, the inventor of the Coolidge tube, Sheareer and Duanne will also read papers. The subject of deep therapy and the production of the hard rays will be fully presented and discussed. The rest of the program will be taken up by a large number of papers on general subjects. The medical profession is cordially invited to attend these meetings.

